# New player in CAR-T manufacture field: comparison of umbilical cord to peripheral blood strategies

**DOI:** 10.3389/fimmu.2025.1561174

**Published:** 2025-03-21

**Authors:** Karolina Rassek, Jan Misiak, Tomasz Ołdak, Natalia Rozwadowska, Grzegorz Basak, Tomasz Kolanowski

**Affiliations:** ^1^ Institute of Human Genetics, Polish Academy of Sciences, Poznan, Poland; ^2^ FamicordTx, Warsaw, Poland; ^3^ Polish Stem Cell Bank (PBKM), Warsaw, Poland; ^4^ Department of Hematology, Transplantation and Internal Medicine, Medical University of Warsaw, Warsaw, Poland

**Keywords:** CAR-T cells, umbilical cord blood, cord blood bank, allogeneic therapy, autologous therapy, off-the-shelf CAR-T cells

## Abstract

One of the most successful treatments in hematologic cancer is chimeric antigen receptor (CAR)-T cell-based immunotherapy. However, CAR-T therapy is not without challenges like the costly manufacturing process required to personalize each treatment for individual patients or graft-versus-host disease. Umbilical cord blood (UCB) has been most commonly used for hematopoietic cell transplant as it offers several advantages, including its rich source of hematopoietic stem cells, lower risk of graft-versus-host disease, and easier matching for recipients due to less stringent HLA requirements compared to bone marrow or peripheral blood stem cells. In this review, we have discussed the advantages and disadvantages of different CAR-T cell manufacturing strategies with the use of allogeneic and autologous peripheral blood cells. We compare them to the UCB approach and discuss ongoing pre-clinical and clinical trials in the field. Finally, we propose a cord blood bank as a readily available source of CAR-T cells.

## 1 Introduction

One of the most widespread approaches in cancer immunotherapies is chimeric antigen receptor (CAR)-T cell therapy, in which genetically modified allogeneic or autologous T cells or natural killer (NK) cells are redirected against the tumor cells armored with CAR construct (1). The classic CAR combines antigen-binding domains, most commonly a single-chain variable fragment derived from antibody variable domains, with the signaling domains of the T cell receptor 3ζ chain and additional costimulatory domains from receptors such as 4‐1BB, OX40, or CD28 (2). In recent years, CAR-T cell therapy has revolutionized the treatment of relapsed or refractory hematological malignancies, resulting in Food and Drug Agency (FDA) approval of six products targeting non-Hodgkin lymphomas (NHLs), acute lymphoblastic leukemia (ALL) and multiple myeloma (MM).

Despite the enormous progress in the treatment, the therapies are associated with several limitations, such as manufacturing difficulties, risk of relapse, or the impossibility of generating clinically applicable doses of CAR-T cells from previously treated patients. Moreover, there are two main risks associated with the use of autologous CAR T cell therapy: cytokine release syndrome (CRS) and neurotoxicity (3). On the other hand, allogeneic CAR-T cells raise safety concerns, as the infusion of donor-derived cells may cause graft-versus-host disease (GvHD) or trigger graft rejection (4).

An appealing strategy to generate “off-the-shelf” allogeneic CAR-T cells with limited potential of triggering GvHD is to utilize umbilical cord blood (UCB) as a source of T/NK cells. To date, UCB has been successfully used as a hematopoietic stem cell (HSC) source in allogeneic HSC transplantations (alloHSCT) with lower rates of GvHD in transplant recipients compared to conventional sources (bone marrow or mobilized stem cells) (5). Crucially, the UCB alloHSCT procedure permits higher human leukocyte antigen (HLA) disparity between a donor and a recipient (6). These qualities are attributed to the naïve phenotype of cord blood T/NK cells (5, 7).

In this review, we discuss the pros and cons of different cell sources for adoptive CAR-T cell immunotherapy. We focus on the therapeutic application and summarize the results from the ongoing clinical trials regarding the use of UCB to provide the perspective for future research teams and clinicians interested in this field. Finally, we propose a novel approach to utilizing CB banks for CAR-T cell manufacturing.

## 2 T cell characteristics

One of the key aspects determining CAR-T cell therapy’s effectiveness and long-term remission is the *in vivo* stability of transferred cells. It has been shown that the persistence of CAR-T cells is correlated with the phenotype of the modified T cells and that prolonged detection of CAR-T cells is associated with a better response even in patients with high-grade cancer (8). Moreover, the differentiation stage of adoptively transferred T cells affects their proliferation and survival which strongly correlates with their antitumor activity (9). Depending on the stage of cell differentiation, T cells can be divided into naïve T cells (TN), stem cell memory T cells (TSCM), central memory T cells (TCM), memory effector T cells (TEM), and effector T cells (TEF) (10).

**Figure 1:**
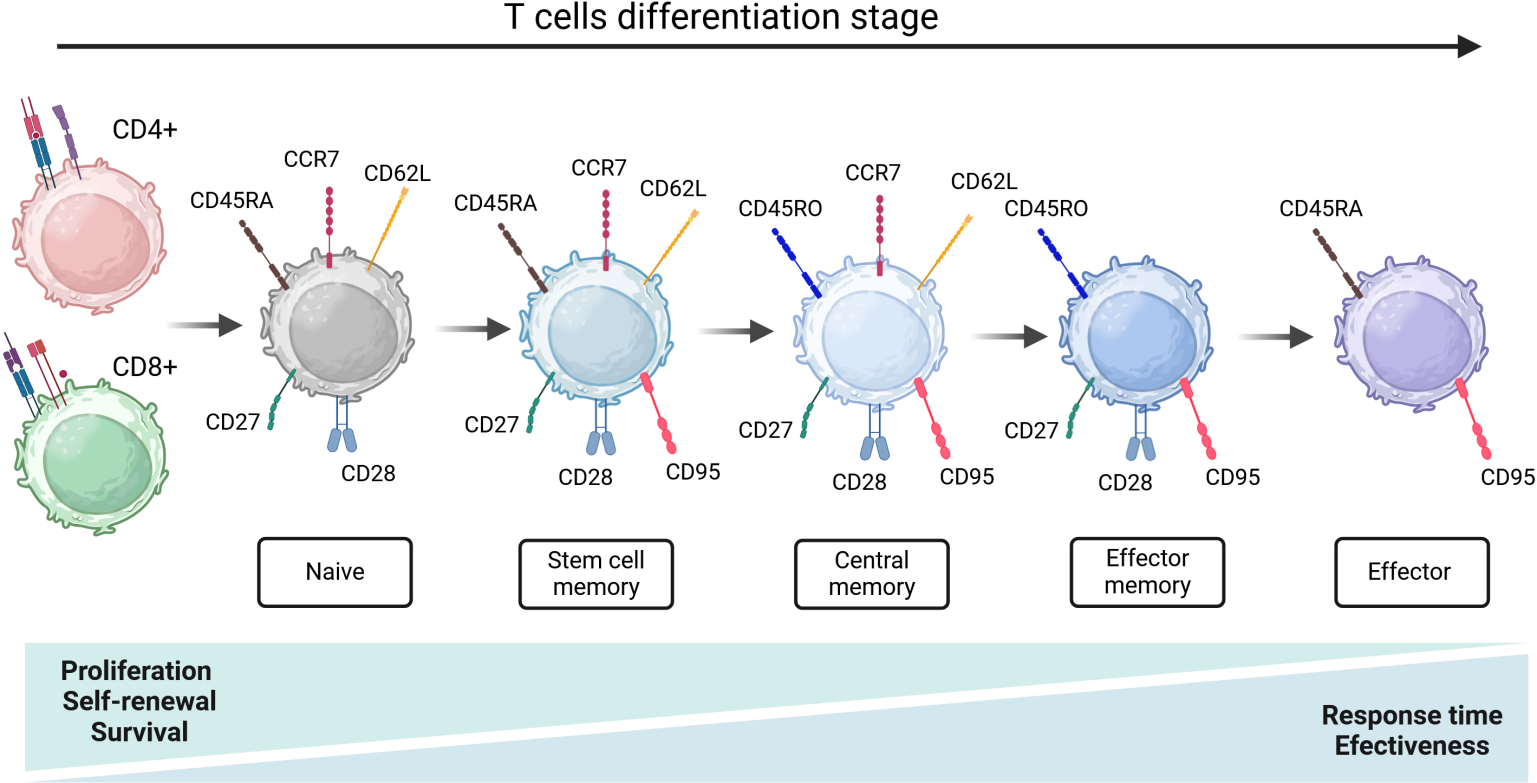
Linear model of stages and characteristics of T cells maturation. Following activation, naïve T cells differentiate into memory and effector cells. This differentiation is marked by the dynamic changes in the expression of CD45RA/RO, CCR7, CD28, CD62L, and CD95 antigens. Created in BioRender.

Naïve T lymphocytes are characterized by high proliferative ability with the surface expression of CD62L, CCR7, and CD45RA isoform and a lack of activation markers like CD25, CD44, CD69, or CD45RO isoform (11). Lymphocytes are considered naïve until they are presented with an antigen that activates TN lymphocytes to proliferate and differentiate into (memory T cells) TCM and/or TEM lymphocytes (12). TSCM is a subpopulation of the least differentiated of the memory T cell subset. Surface markers expressed by TSCM include both naïve T cell markers, such as CD45RA, high levels of CD27, CD28, CD127, CD62L, and CCR7, as well as markers of memory T cells, such as CD122, CD95, or CXCR3 but do not include CD45RO (13, 14). They have stem cell-like self-renewal capacity and can regenerate all TCM and TEF cell populations (15, 16). Compared to other subpopulations, the T subset of TSCM develops a faster response to antigenic stimulation and may persist for a long time (12). TCM cells guard lymph nodes, providing central immunosurveillance against known pathogens. Compared to naïve T cells, memory T cells respond more quickly and deliver an early batch of cytokines when stimulated by a specific antigen (17). TCM secrete tumor necrosis factor α (TNF-α), interleukin 2 (IL-2), and co-express L-selectin and CCR7, as well as CD45RO, CD62L, CD27, but not CD45RA (18). Next population - TEMs produce many inflammatory cytokines such as TNF-α and interferon γ (IFN-γ) as their most important role is related to the activation of the immune response taking place in peripheral lymphoid organs (19). Phenotypically, TEM cells are positive for activation markers CD38, CD69, and CD25, they sometimes co-express CD45RA and CD45RO at high levels, however, are negative for CD62L, CCR7, and CD31 (20). Finally, the TEF subpopulation contains fully differentiated T cells, responsible for strong effector functions (12). TEF overexpress several homing receptors to migrate to inflammation sites like CCR5, LFA-1, as well as CD45RA, CD95, CD122, and KLGR1 but do not express CD45RO, CCR7, CD27, CD62L or CD28 (21, 22). Additionally, TEFs exhibit limited expansion, low self-renewal, and survival capacity as a consequence become exhausted quickly (23) (**Figure 1**).

Naïve CD4+ T cells differentiate into several effector T cell subsets characterized by the ability to produce specific cytokines, namely helper T cells (Th) Th1, Th2, Th9, Th17, Th22, follicular helper T cells (Tfh) and regulatory T cells (Treg). Cell differentiation is regulated by the cytokines present in the environment and induced upon T cell receptor (TCR) signaling (24). Foreign antigens from infected or unknown cells are presented to T cells in the context of specific major histocompatibility complex I (MHC class I) called HLAs. Each cell subset has particular characteristics and releases cytokines that determine its function, such as Th1 and Th2 cells which produce IFN-γ and IL-4, respectively (25). CD4+ T cells are differentiated through the cytokines upon induction by master transcription factors such as T-bet, GATA-3, RORγt, Bcl6, or Foxp3 (26).

Studies showed that both CD4+ and CD8+ CAR-T cells derived from TCM and TM populations can be involved in killing malignant cells and achieve better results than those from TEM (27). Results from the clinical trials indicate that CAR-T cell products rich in TN, TSCM, or TCM are required for the sustained *in vivo* persistence of adoptively transferred CAR-T cells due to their ability to proliferate (28, 29). Moreover, the combination of the most potent CD4+ and CD8+ CAR-T cell subsets has a synergistic antitumor effect *in vivo* (30). These results demonstrate that naïve and TSCM/TCM cells are the most important players in CAR-T cell therapy due to their sustained proliferation and persistence *in vivo*.

Comprehending the phenotype of manufactured CAR-T products is critical to mitigate adverse events, as well as to improve antitumor effectiveness, such as CAR-T dosing or activation. The phenotypic profile of infused CAR-T cells has not only been linked to the cells’ persistence but also to the long-term outcomes of the treatment (18, 31).

## 3 CAR molecular constructs

CARs are created using the main signaling components of the T cell receptor and costimulatory molecules. In general, CAR consists of the extracellular single-chain variable fragment (scFv) specific for a cancer marker, and three main domains: an extracellular antigen-binding domain derived from a tumor-specific monoclonal antibody, a transmembrane domain that anchors the CAR to the T cell (derived from CD3, CD4, CD8 or CD28 proteins) and an intracellular T cell activation domain of CD3ζ with one or more costimulatory domains (usually from 4–1BB or CD28) that is required for full T cell activation (32–35).

Since the introduction, several CAR-T cell generations have been developed. First-generation CARs had only one signaling domain, typically the CD3ζ that proved not to be sufficient due to the limited persistence and signaling ability of the cells (36). To improve that, the second generation of CARs included a co-stimulatory signaling domain (usually CD28 or 4-1BB) localized proximal to the membrane to enhance activation, and survival, and promote the expansion of the modified T cells (37). To achieve greater antitumor activity and increase CAR-T cell persistence, a second co-stimulatory signaling domain (e.g. CD28 or OX40) was incorporated into third-generation CARs (38). The fourth generation of CARs also known as “TRUCKs” (T cells redirected for universal cytokine killing) was based on the structure of the second-generation CARs. TRUCKs contain a transgenic expression cassette coding for a synthetic nuclear factor of activated T cell response elements to be produced and secreted upon antigen recognition, such as IL-12 (39). The secretion of cytokine enhances the immune response against cancer cells and recruits other immune cells to the tumor site (40). The next generations of CARs are often based on the second generation and contain several modifications including, truncated intracellular domains of cytokine receptors such as IL-2 with the addition of STAT3/STAT5 transcription factors binding motifs (41). These CAR constructs induce cytokine secretion through the activation of the JAK/STAT signaling pathway therefore driving CAR-T cells to remain active and generate TM cells (42).

**Figure 2:**
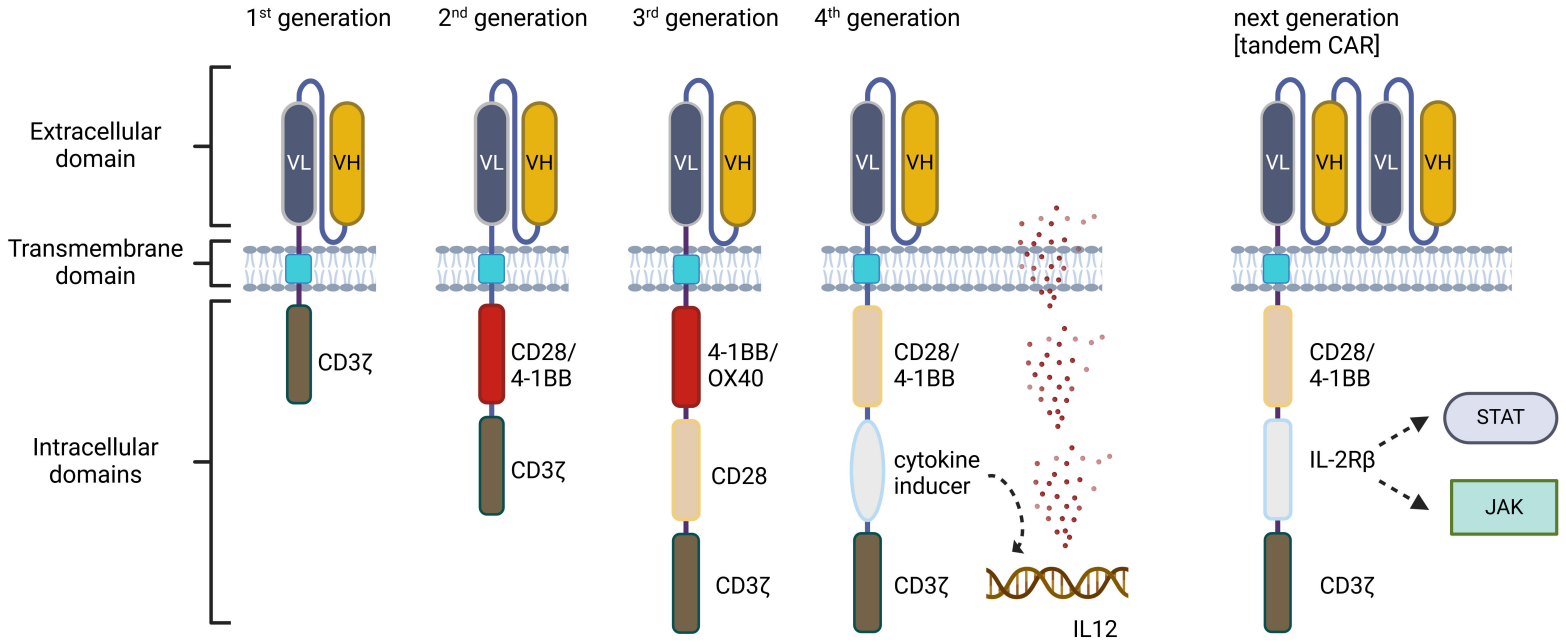
Generations of chimeric antigen receptors (CARs). The first generation of CARs is equipped with a single CD3ζ intracellular domain. The second generation includes additional costimulatory molecules, such as CD28 or 4-1BB. The third generation combines multiple co-stimulatory domains, like CD28-4-1BB or CD28-OX40. The fourth generation builds upon second-generation CARs, incorporating either constitutively or inducible expressed chemokines. The next generation of CARs retains the structure of the second generation but also incorporates cytokine receptors in the intracellular domain, such as the IL-2Rβ chain fragment. VL, variable region of light chain; VH, variable region of heavy chain. Created in BioRender.

Novel developments of CAR constructs are still being extensively studied and include logic gating with the example of tandem CAR-T cells targeting two antigens simultaneously or SynNotch-engineered CAR-T cells that use a synthetic Notch receptor to sense a specific antigen, which then triggers the expression of the CAR and many others CAR variants and augmentations (43, 44) (**Figure 2**). On the other hand, extensive clinical experience showed that overactivation of the peripheral T cells leads to extensive exhaustion and might not be the only possible way of CAR therapy development in the future. Genome editing is also being explored to improve next-generation CAR-T cells to overcome some of the current limitations of the therapy. To improve CAR-T cells’ persistence and function both knock-out/knock-in genes as well as epigenetic modifications are being extensively studied (45).

While scientists and industry continue their work on novel generations of more persistent and capable CAR-T cells, the genetic modification of constructs involves certain risks and challenges, both in the laboratory during the genetic engineering process and in the patient’s body after the CAR-T cell transfer. One of the concerns relates to the safety of viral vectors. The use of viral vectors (lentiviruses or retroviruses) to deliver the CAR gene into T cells carries a risk of unintended genetic mutations or insertional oncogenesis, where genetic modification leads to the development of cancer (46). Although the FDA has called to label CAR-T cell products with a warning about secondary T cell mutagenesis, insertional mutagenesis was never proven to be a cause (47). Moreover, a recent study analyzed retrospective data from 61 centers worldwide including 3038 B-cell and non–B-cell malignancies treated with commercial or investigational CAR-T cell products in children, adolescents, and young adults. Remarkably, no cases of T-cell malignancies were reported following CAR-T cell treatment in this cohort (48).

Another risk applies to the off-target effect. Despite CAR-T cells being designed to target specific antigens on cancer cells, there is a risk of unintended attack of an antigen other than the intended one or activation of the CAR-T cells independently from their specificity (49).

## 4 Autologous CAR-T therapy

Autologous CAR-T cell therapy, where the patient’s T cells are genetically engineered *in vitro* to express the CAR has proved its importance in clinical applications (50). Since the therapy uses the patient’s T cells, it is highly personalized and tailored to the person’s immune system. This reduces the risk of GvHD, a common complication of allogeneic stem cell therapy or CAR-T cell therapy that uses donor cells (51). Additionally, very high response rates, even in patients with limited treatment options followed by highly durable remissions have been observed after CD19-targeted CAR-T cell therapy (52–54).

However, this approach is not without drawbacks. Firstly, as it relies on autologous T cells, it depends on the nature and quality of T cells in the peripheral blood (PB) of the patient at the time of treatment. Frequently, patients have compromised immune systems and suffer from severe lymphopenia as a result of previous treatments which affects the quality and quantity of T cells harvested during apheresis (55). Besides, harvested T cells may have impaired functionality, reduced proliferative capacity, and altered phenotypes impacting their usefulness for CAR-T cell manufacturing (56).

A therapeutic dose of 10^7^ – 10^9^ cells is required for the treatment, hence too low T cell count or cell activation failure during the production process may be the cause of treatment failure. What is more, patient cells can be contaminated with malignant tumor cells (57). Autologous CAR-T cells may be also exposed to the patient’s tumor microenvironment before infusion. This leads to immune exhaustion, where CAR-T cells display decreased proliferative capacity, diminished anti-tumor activity, and attenuated persistence (58). Another issue is the high manufacturing cost of CARs due to the complex and personalized manufacturing process as autologous cell therapy is “tailor-made” meaning that that CAR-T cells must be generated *de novo* for each patient (59). Factors such as the time, materials, infrastructure, and skilled manpower required to generate CAR-T cells for each patient contribute to the astounding therapy cost. Additionally, as one batch is custom-made for one patient, the economy of scale cannot be applied (60). Time is another very important issue. In many cases, CAR-T cell therapy is the last treatment option for severely ill patients. In the case of autologous treatment, the production of personalized CARs requires a long manufacturing process. To successfully carry out the entire process of transporting a patient to the manufacturing facility and back to the treatment facility requires a thorough complex transport network, planning, and a skilled workforce that is highly prone to delays. This can result in an increase in treatment time of three to five weeks, which may be intolerable due to the progression of the patient’s disease (61). Recent pre-clinical studies developed a new platform called FasT CAR-T with a shortened next-day manufacturing time. The procedure was reported successfully with a median of 14 days from apheresis driven by 7 days of rapid sterility testing, which is a significant improvement from the median of 45 days. Although the first-in-human clinical study showed early promising efficacy in B-ALL patients, more data on additional patients and longer follow-up are needed to further evaluate the efficacy of this novel CAR-T cell therapy (62, 63). Lastly, while CAR-T cell therapy has demonstrated clinical efficacy in hematologic malignancies, severe toxicities such as CRS have also been reported (64). CRS is characterized by the release of cytokines (mainly IFN-γ, IL-6, IL-10, TNF-α, and IL-2) in response to the therapy. That causes systemic inflammatory responses resulting in flu-like symptoms and, in severe cases, organ dysfunction (65). The precise characterization and understanding of the composition of the infused T cells both in terms of T-cell memory differentiation and CD4/CD8 ratio are fundamental aspects of CAR-T cell therapy development and implementation. Collectively, directly impacts the antitumor activity, safety, and consistency, ultimately leading to better therapeutic outcomes (30, 66).

## 5 Allogeneic CAR-T therapy

Donor-derived allogeneic CAR-T cell therapies offer many advantages compared with autologous treatments. One of the benefits of the allogeneic approach is the possibility of obtaining a higher quality of donor cells, which directly translates into the quality of the treatment (67). Healthy donors can be pre-screened for desirable characteristics of T cells like an appropriate number, phenotype, or optimal CD4:CD8 ratio that will minimize manufacturing errors. Selection of donors that have a high percentage of T cells is recommended, as contamination with non-T cell populations may affect downstream production (68). In contrast to autologous products, a large number of allogeneic CAR-T cells can be generated in a single manufacturing run making the production process cost-effective. Moreover, during production, standardized T-cell products from healthy donors are being created ahead of time, making it possible to generate many therapeutic doses from one manufacturing lot making it readily available for patients (69). Allogeneic CAR-T cells as “off-the-shelf” can reduce “vein-to-vein” time, decrease the risk of manufacturing failures, and alleviate logistical challenges (69). Eliminating treatment delays by reviving pre-frozen allogeneic CAR-T cells whenever the patient needs them may also improve the clinical outcomes of the therapy (70). Moreover, allogeneic CAR-T cell therapy does not carry the risk of contamination with cancer cells as T cells are manufactured from the healthy donor’s blood. Allogeneic CAR-T cells may also have a lower risk of immune exhaustion as they are not exposed to the patient’s tumor microenvironment or cytotoxic therapies prior to infusion (71).

Although allogeneic CAR-T cell therapy has numerous advantages, one of the most significant drawbacks is the risk of the development of GvHD (64). A condition where the T cell αβ receptor (TCRαβ) on the infused CAR-T cells recognizes cell surface HLA class I and class II molecules on the recipient’s cells and attacks them. Therefore, ensuring compatibility between the donor and recipient’s HLA types is essential to reduce the risk of GvHD, which may limit the availability of suitable donors (72). Until now, it has been established that for successful HLA-matching, the patient and potential donor should have high-resolution HLA typing using DNA-based methods for HLA-A, B, C, DRB1, and DQB1 (73).

Recently studies proposed a feasible alternative for HLA match assessment in terms of CAR-T cells derived from PB T cells. The higher efficacy of HLA-matched CAR-T cells (minimum of a 4/6 match to the patient) compared to HLA-haploidentical (sharing one haplotype) CAR-T cells has been observed (74). Additionally, results showed that haploidentical PB CAR-T cells induced only transient or no reduction in PB leukemia cell number without significant CAR-T cells expansion, which suggests rejection (74).

Achieving HLA compatibility is crucial not only to reduce the risk of GvHD but also to prevent CAR-T cell rejection. For that reason, patients receiving allogeneic CAR-T cells often require immunosuppressive drugs, which can weaken the anti-cancer immune response and increase susceptibility to infections (69). Hence, genome editing has been applied to prevent GvHD. Studies showed that the elimination of expression of the endogenous TCRαβ receptors using either zinc finger nucleases (ZFNs) or transcription activator-like effector nucleases (TALENs) successfully prevented GvHD without compromising CAR-dependent effector functions (75, 76). In two phase 1 clinical trials in pediatric and adult patients with late-stage relapsed or refractory B-cell acute lymphoblastic leukemia (NCT02808442 and NCT02746952), TALENs were used to simultaneously disrupt cell surface expression of TCRαβ and CD52. Results proved that genetically modified CAR-T cells caused only minimal GvHD with no detrimental expansion of unedited TCRαβ-positive T cells (77). Another phase 1 study (NCT04227015) has utilized the alternative genome editing technique CRISPR/Cas9 to disrupt the TCRα subunit constant (TRAC) region previously shown to lead to loss of TCRαβ expression (78). Obtained data demonstrated that CD19/CD22-targeting CAR-T cells with a CRISPR/Cas9-disrupted TRAC region and CD52 gene infused in patients with relapsed/refractory acute lymphocytic leukemia showed manageable safety profile and notable antileukemia activity, with no GvHD observed (79).

Nevertheless, complications and toxicities may arise as a result of gene editing. This may include on- and off-target effects and issues such as CRS or neurotoxicity, especially when editing inadvertently causes the CAR-T cells to become more aggressive or activate faster than desired (80). Another challenge of allogeneic therapy combined with more extensive genetic modification is the risk of chromosomal aberrations. No currently available manufacturing process can provide 100% certainty that no aberrations are present in a therapy’s potentially hundreds of millions of cells. In 2021, the FDA put a 4-month hold on clinical trials of all allogeneic projects due to chromosomal abnormality found in a lymphoma patient in the Alpha-2 trial of the CD19-directed allogeneic CAR-T trial. Although the investigation has not shown any clinical consequences, such as aberrant proliferation or leukemogenesis of engineered cells, the issue of chromosomal aberrations risk is to be addressed (81).

CAR-NK cells are being investigated as an alternative cell source in allogeneic cell therapies as they are crucial in immune surveillance of invading viruses and killing cancer cells without the need for tumor-specific antigen presentation (82). Except for the CD3 signaling domain, as well as CD28 or 4-1BB as a co-stimulatory domain, CAR-NK cells enhance their cytotoxic capacity and cytokine production through additional co-stimulatory molecules, that is: NKG2D and CD244 (2B4) (83). The initial success of NK cell therapies has prompted further investigation into current allogeneic NK cell products, yielding encouraging outcomes in clinical trials. First, since NK cells do not recognize targets presented by the HLA system, therapeutic CAR-NK cells can be cultivated from donors with various genetic backgrounds and applied in diverse recipients without the concern of inducing GvHD (84). Second, due to a different spectrum of secreted cytokines, CAR-NK therapy does not cause CRS, therefore it is considered safer than CAR-T cells treatment (85). Third, NK cells are characterized by the abundance of CD16 (FcγRIIIA), which serves as a receptor for IgG1 and IgG3 and is essential for NK cell-mediated antibody-dependent cell-mediated cytotoxicity ADCC (86). Hence, they can also productively eliminate tumor cells in a CAR-independent manner through their stimulatory and inhibitory receptors which form the basis for the dual anti-tumor activity of CAR-NK cells.

In recent years several studies explored different approaches to genetically engineer CAR-NK cells. Especially in the treatment of T-ALL CAR-NK cell therapies targeting CD3, CD5, and CD7 have shown significant anti-tumor cytotoxicity both *in vitro* and *in vivo* (87–89). As of January 2025 currently ongoing clinical trials on CAR-NK focus not only on CD19 target but also on CD123 *clinical trials.gov; ID* (NCT05574608) and *clinical trials.gov; ID* CLL-1(NCT06307054) in acute myeloid leukemia (AML) or dual targeting of CD19 and CD70 *clinical trials.gov; ID* (NCT05842707) in B cell Non-Hodgkin lymphoma and CD33 and/or FLT3 in AML *clinical trials.gov; ID* (NCT06325748).

As pre-clinical and clinical data support the potential of CAR-NK cell therapies, still several challenges need to be addressed before clinical application. One of the main barriers to CAR-NK cell therapy is the lack of persistence *in vivo* in the cytokine support deficiency (90). Additionally, due to the inhibitory tumor microenvironment, CAR-NK ability of homing and infiltration is limited (91).

## 6 UCB as a source for CAR-T cells

### 6.1 Characteristics of UCB as cells source

**Figure 3:**
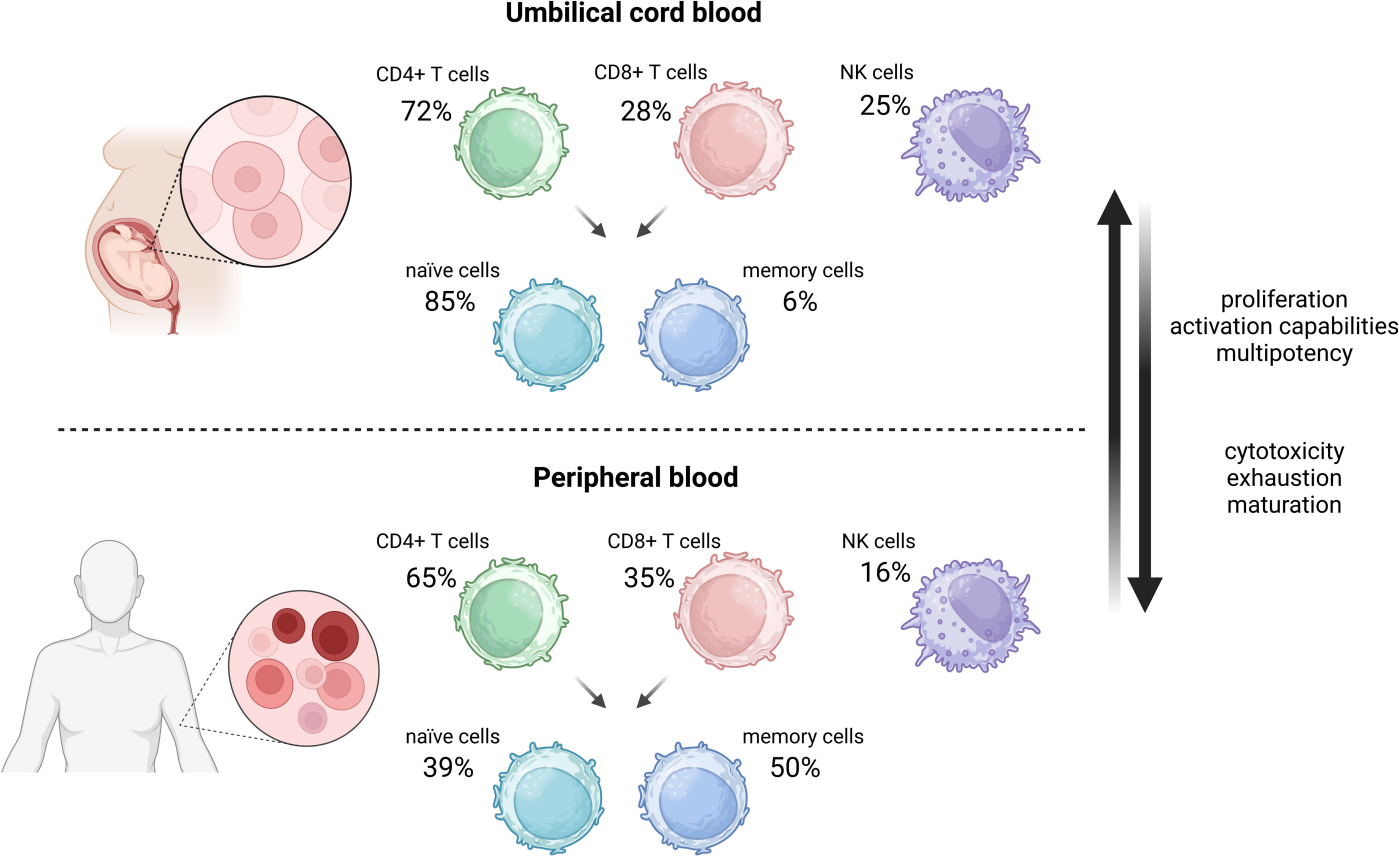
Comparison of umbilical cord blood and peripheral blood cells composition in terms of CAR-T cells application. Created in BioRender.

Over the past years, UCB transplantation has become an alternative therapeutic option for patients with cancer or genetic diseases for whom an HLA-matched family or unrelated adult PB stem cells or bone marrow donor has not been found (92). UCB therapy is currently used for the treatment of such hematological diseases as myelodysplastic syndrome (MDS), AML, aplastic anemia (AA), or ALL (93–96). It is the unique qualities of UCB compared to PB or bone marrow that make the therapy encouraging. In contrast to PB cells, UCB cells are characterized by higher proliferation, and expansion due to the higher percentage of hematopoietic stem cells, with the less mature phenotype of T cells and NK cells (97, 98). Crucially, the UCB T-cell population is characterized by a more naïve phenotype as it comprises approximately 85% naïve CD45RA^+^/RO^-^ T cells and only 6% memory T cells in comparison to 39% and 50% in PB, respectively (99) (**Figure 3**). Remarkably, naïve T cells of UCB differ from PB naïve T cells (100, 101). UCB naïve T cells have higher proliferative and activation capacities, as well as delayed exhaustion compared to PB (100). They do display comparable activation levels with reduced secretion of cytokines such as IL-2, IFN-γ, and TNF-α upon CD3/CD28 targeted stimulation. Moreover, naïve UCB T cells are characterized by lower expression of CD40L and perforin thus, they exhibit lower cytotoxicity than PB cells. Furthermore, compared to PB, UCB contains a relatively high frequency of distinct CD4+CD25 bright subset of regulatory T cells. Thus, the likelihood of co-purification of activated or memory CD4 + 25+ T cells in UCB compared with PB is decreased. Last but not least, UCB cells display elevated self-renewal potential as well as multiple cell divisions attributed to longer telomeres and overexpression of transcription factors like NF- κB (102).

Therefore, one of the major advantages of UCB treatment is its safety. Compared to other donor cell resources, UCB collection not only painless but also carries a lower risk of viral infections and somatic mutations, which are the primary causes of morbidity following transplantation (103). Moreover, in the case of allogeneic transplantation, UCB allows for less stringent HLA matching criteria (4 of 6 HLA loci, considering a low resolution for HLA-A and HLA-B and high resolution for HLA-DR), which may be especially important for members of racial and ethnic minorities, increasing the inclusiveness of the therapies (104). UCB grafts could also reduce the mortality of transplant as due to its low immunogenicity decrease in both acute and chronic rates of GvHD are observed (105). Studies showed that PB consists higher concentration of CD8+ T cells comparing to UCB (97, 106). Given that GvHD is primarily driven by alloreactive donor CD8+ T cells, this difference in composition may help to explain the higher frequency of the chronic GvHD observed for example when the PB is used as a stem cell source for hematopoietic stem cell transplantation (107). Additionally, PB contains more mature monocytes, lower counts of plasmacytoid dendritic cells (pDCs), and reduced numbers of NK 56bright16− cells, further contributing to increased GvHD risk, when compared to UCB (108). At a molecular level, UCB T cells exhibit impaired nuclear factor of activated T cells (NFAT) signaling as well as lower activation of the NF-κB pathway that leads to reduced production of several pro-inflammatory cytokines, further decreasing GvHD frequency and intensity (109–111). On the other hand, there are several drawbacks to UCB cell usage. To begin with, in the case of autologous transplant and treatment of cancer or genetic disorders, stem cells derived from UCB may not be suitable as they might encompass the same abnormal cells or genetic variants that caused the onset of the disease at first (112). Furthermore, very often the amount of stem cells harvested from UCB is insufficient for the treatment of adults (113, 114). The last concern is the longer engraftment of CB stem cells (21-25 days) compared with adult donor cells (20 days) associated with higher hospital costs (115). To overcome the obstacles mainly related to the limited number of cells available in a single unit, several approaches have been investigated, including double UCB transplantation, *in vitro* UCB culture expansion, or direct intrabone transplantation of UCB cells (116–118).

Nowadays, haploidentical (haplo)-cord transplantation represents a promising approach to allogeneic transplantation. This technique involves combining two stem cell sources: UCB and CD34^+^ cells from haploidentical donors (119). Haplo-cord transplantation has shown several benefits such as rapid availability and less stringent HLA-matching requirements compared to unrelated traditional adult grafts as it combines the benefits of both haploidentical and UCB transplants (120). Since the haploidentical component is derived from a partially matched family member and the UCB component relies less on perfect HLA matches because of its naïve immune cells, the two stem cell sources complement each other and yield better outcomes than using either source alone. Furthermore, this approach is associated with accelerated neutrophil and platelet recovery, lower risks of acute and chronic GVHD, and excellent graft-versus-leukemia (GVL) effects (121). Additionally, when combined with reduced-intensity conditioning (RIC), haplo-cord transplantation has shown decreased susceptibility to delayed opportunistic infections, shortened hospital stays, and favorable long-term outcomes (122).

### 6.2 Hurdles in UCB use as a CAR-T cell source

As UCB T cells exhibit distinct naïve profiles, they recently became an attractive source for CAR-T cells as an alternative to autologous cell treatment, potentially broadening access to the therapy. UCB as a CAR-T cell source offers advantages such as the shift of CD4^+^25^+^ T cells toward a Treg suppressor cell phenotype that may contribute to a lower incidence of GvHD after CAR-T cell infusion (123). Furthermore, UCB cells express significantly lower markers of exhaustion such as programmed cell death-1 (PD-1), T-cell immunoglobulin, and mucin domain-containing protein-3 (TIM-3) and lymphocyte activation gene-3 (LAG-3) compared to PB T cells, allowing for long-term persistence and efficiency (124, 125). However, the use of UCB cells for CAR-T therapy raises several challenges. Firstly, due to their naïve state, UCB-derived CAR-T cells may require longer expansion time. While studies have reported successful generation of CAR-PB-derived CAR-T cell products in as little as 8 days, UCB-derived CAR-T cells may need more time to achieve comparable therapeutic dose (126, 127). It is unclear for now how the “fast-CAR” approaches would be working in UCB case as well. What is more, specific phenotype distribution plays a significant role in establishing the proliferative and expansive potential of UCB-derived cells (126). Therefore, the specific phenotype of naïve UCB-derived T cells must be considered when designing and interpreting UCB CAR-T studies. These include protocols for activation and cytokine stimulation in the manufacturing process, the ability to differentiate into effector cells, and cytotoxicity. Current manufacturing of CAR-T products involves induction of cell activation and expansion by targeting CD3/CD28 molecules and coincubation with interleukin (IL) 2 or IL-7 and IL-15. Notably, it is reported that IL-2 stimulation may cause cellular exhaustion, whereas IL-7 and IL-15 contribute to a higher percentage of memory cells. However, this evidence applies to conventional, PB-derived T cells so a more tailored and detailed study of UCB-derived T-cell activation pathways should be performed (128). Additionally, there is a question of whether the manufacturing process could deprive UCB T cells of their naivety features while providing them with sufficient anti-tumor cytotoxicity. According to studies, including our data, cord blood T cells retain a phenotype similar to T cells derived from peripheral blood upon CD3/CD28 stimulation and cytokine priming (129). Some differences could be attributed to the lower expression of exhaustion markers, nevertheless, the cytokine repertoire used in the manufacturing process significantly contributes to the phenotype of the resulting CAR-T cells (126).

Another concern is whether UCB-derived CAR-T cells could perform effector functions. Since T cells from UCB are more naive and less experienced in fighting infections or cancer compared to T cells from adult donors, it may take longer to adapt and respond effectively to cancer cells, potentially delaying the therapeutic effect. The seminal study completed by Serrano et al. proved that these cells are capable of CD19-specific cytotoxicity and was followed by results from other research groups (126, 130). However, in parallel with reports of satisfactory anti-tumor efficacy, concerns regarding adverse effects in allogeneic settings arise. Moreover, as UCB contains a relatively small number of T cells compared to PB, this limited T cell quantity may be insufficient for generating a therapeutic dose of CAR-T cells, especially for adult patients or those with high tumor burdens. Therefore, *ex vivo* expansion may be required to proceed with cellular transfer (131). The efficiency of the viral transduction, a crucial step in CAR gene introduction is yet another concern. Studies have shown variable transduction rates between units (15-85%), possibly due to T cell differentiation profiles (126, 132).

Besides the hurdles, several studies showed the feasibility and great therapeutic potential of UCB-derived CAR-T as such. Augmentation of attributes with targeted reduction of defective features should be in focus of new adoptive therapy approaches. **Table 1** provides a comprehensive comparison of PB-derived and UCB-derived CAR-T therapies, highlighting key differences in aspects such as time, cost, cell quality, and risk profiles across autologous and allogeneic approaches.

**Table 1:** Comparison of different approaches to generate CAR-T cells.

	PB-DERIVED THERAPY		UCB-DERIVED THERAPY
	AUTOLOGOUS THERAPY	ALLOGENEIC THERAPY	
TIME	3-5 weeks; cells generated *de novo*	“off-the-shelf” product; can be generated in advance	can be generated in advance
COST	more expensive	cost-effective	depends on availability of suitable cord blood or tissue samples and the cost of processing and expansion
QUALITY OF CELLS	T cell count may not be enough, cell activation failure, contamination with malignant tumor cells	cells pre-screened for appropriate number, phenotype, or optimal CD4:CD8 ratio	readily available, high quality, and immunologically compatible hematopoietic cells
CUSTOMIZATION	highly personalized and tailored to the person’s immune system	“universal” donor cell lines	broad application
QUALITY CONTROL	variability in T cell quality and function based on individual patient factors	manufactured under controlled conditions, ensuring consistency and quality across multiple batches	FDA regulated product
APPLICABILITY	not applicable in patients with poor T-cell parameters	broad application; can be used in patients with poor T-cell parameters	broad application both in autologous and allogeneic therapy
T-CELL EXHAUSTION RISK	common	lower risk of immune exhaustion	lower than with autologous cells
GRAFT VERSUS HOST DISEASE RISK	none	common	minimal

PB, peripheral blood; UCB, umbilical cord blood; FDA, Food and Drug Administration.

### 6.3 Preclinical reports on UCB CAR-T safety and efficacy

As the immunology of UCB-T cells supports their use as a source for CAR-T manufacturing, several both *in vitro* and *in vivo* strategies have been tested in preclinical settings worldwide.

First of all, it has been proven that UCB-T cells cells can be successfully engineered to express CAR receptors capable of recognizing specific markers (133). Serrano et al. first reported that naïve UCB-T cells cells could be engineered into CD19 CAR-T cells (130). Encouraging results showed a reduction in tumor bulk in the *in vivo* arm of the study, with 60% of mice achieving complete remission with no adverse reactions following CAR-T infusion observed. Later, Huang et al. engineered 2^nd^ generation anti-CD19 product from UCB with what seems to be an exquisite transposon-based mechanism (134). *In vitro* assays on B-cell lymphoma and B-cell ALL cell lines showed the efficient killing of tumor cells by UCB-derived CAR-T cells. The researchers also showed that UCB-derived CAR-T cells were capable of short-term (10 days) anti-tumor efficacy *in vivo* regardless of IL-2 injections. Nevertheless, there was no mention of off-target toxicities (134). Notably, in a direct comparison between CAR-T cells manufactured from PB, UCB-derived CAR-T cells presented similar anti-tumor responses completely eradicating B-cell ALL blasts over 5 days of coculture in the *in vitro* assay (135).

Another study of UCB CAR-T cells targeting B-cell ALL was conducted by Liu et al. The researchers compared the efficacy of autologous CAR-T cells and donor-derived, either PB or UCB CAR-T cells (136). Unsurprisingly, both donor-derived PB and UCB CAR-T cells showed increased proportions of cells displaying an immature phenotype (including naïve T cells and central memory T cells) as well as induced better responses *in vivo* than ALL patient-derived CAR-T cells. The survival analysis showed that the mice in the CB CAR-T and PB CAR-T groups survived longer than those in the PB (patient) CAR-T group with the median survival times of the CB CAR-T, PB CAR-T, and PB (patient) CAR-T groups 51 (39–51) days, 51 (46–51) days, and 32 (25–33) days (p < 0.05), respectively. Moreover, CAR-T and tumor cell proportions in PB revealed that on days 14 and 28, mice in the UCB CAR-T and PB CAR-T groups had higher CAR-T cell expansion and lower tumor burden compared to those in the ALL CAR-T group, making it a promising source for allogeneic CAR-T cells.

Addressing the choice of the most suitable costimulatory domain for UCB-derived CAR, the study Yu et al. elucidated the anti-tumor capacity of UCB-derived CAR with 4-1BB co-stimulatory domain (137). Obtained results showed the target-specific killing of CD19+ T cell lymphoma cells *in vitro* (over 50% dead cells in 1:1 ratio up to 85% dead cells in 10:1 ratio) as well as inhibited tumor progression *in vivo* (<1000 mm3 vs >1500 mm3 tumor volume in controls). UCB CD19-CAR- T cells were also associated with minimal GvHD as no diarrhea, rash, or jaundice, which are common symptoms of GvHD, were observed during the observation period. Furthermore, Tammana et al. indicated that incorporating the 4-1BB domain yielded better responses *in vivo* than CD28. The results showed that the construction of 3^rd^ generation CAR-T cells with both (4-1BB and CD28) domains was associated with even more potent anti-tumor response compared to CAR-T cells with single 4-1BB construct in mice CD19(+) leukemia and lymphoma tumor models, suggesting a synergistic role costimulation in engineering antileukemia UCB effector cells. A systemic NOD/SCID mouse model with established Raji tumors showed that UCB T cells expressing both CAR constructs exhibited significantly improved tumor control and reduced bioluminescence intensity compared with 4-1BB CAR and GFP controls, with bioluminescence intensity in the 4-1BB and CD28 accounting for one-third of the value in the 4-1BB group on day 8 (132). A unique approach was adopted by Pegram et al. The researchers constructed a novel anti-CD19 CAR construct (armored CAR), programmed to secrete IL-12 to interfere with the immunosuppressive cytokine profile within the tumor (138). Obtained results suggested that IL-12 is an important factor for phenotypic changes, such as increased CD62L, CD28, GzmB, and IFNγ. In accordance with previous studies, the UCB-derived CAR-T cells showed promising anti-tumor efficacy both *in vitro* and *in vivo* as they enhanced anti-tumor efficacy compared to the CD19 CAR alone. Notably, the transfer of UCB-T cells secreting IL-12 resulted in increased survival of CD19+ tumor-bearing mice (>40 days of survival vs <40 days of survival compared to controls; *P < 0.05) without a need for pretreatment (irradiation) or IL-2 support. Similar results were obtained while analyzing UCB-derived CAR-NK cells. The study by Herrera et al. highlighted the higher anticancer activity of UCB-derived CAR-NK cells in the treatment of primary chronic lymphocytic leukemia (CLL) compared to CAR-NK from adult cell sources. The study showed that UCB CAR-NK cells exhibited a more stable cell count per unit and demonstrated responsiveness to various interleukins to enhance their *in vitro* expansion, tumor cell killing activity, and promote prolonged cellular survival (139).

To date, four studies investigated the efficacy of UCB-derived CAR-T cells against cell lines other than B-cell lineage. Ma et al. investigated *in vitro* efficacy of uncommon HLA-A-targeting CAR T cells in AML (140). The group developed TCR-like monoclonal antibody (8F4) UCB-derived CAR-T cells that specifically recognized the PR1/HLA-A2 on the surface of AML cells and were capable of killing leukemia cell lines and primary AML blasts in an HLA-A2-dependent manner (>60% of killing and >40% of killing respectively in 4:1 effector: target ratio). A more advanced study was completed by Caël et al., who generated anti-CD123 UCB CAR-T cells, compared them with PB counterparts *in vitro*, and assessed the efficacy *in vivo* (126). Using blastic plasmacytoid dendritic cell neoplasm models they proved that UCB-derived CAR-T cell product retains the pool of less differentiated cells after nine days of expansion using IL-7 and IL-15. Notably, UCB CAR-T CD4^+^ T cells exhibited a less differentiated phenotype compared to PB CAR-T cells, with a higher proportion of TSCM and TCM (68.1% vs. 31.8%, p < 0.001) and a lower proportion of TEM and TEMRA (31.8% vs. 68.2%, p < 0.001). While UCB and PB CD8^+^ T cells showed no significant differences, UCB CAR-T cells trended toward a less differentiated profile, with 49.2% vs. 32.8% TSCM + TCM (p = 0.055) and 51.0% vs. 67.1% TEM + TEMRA (p = 0.064). Additionally, UCB-derived CAR-T cells presented comparable efficacy to PB product (94.3 ± 3.8% and 93.8 ± 3.9% cytotoxicity at E:T ratio 1:1 respectively) *in vitro* and contributed to significantly better overall survival (>120 days vs around 40 days in controls; P=0.004) in leukemia model*in vivo*. Moreover, they demonstrated that thawed or fresh UCB as a source for CAR-T manufacturing does not affect the product’s functionality. In a study conducted by Pinz et al., 3^rd^ generation UCB CAR-T cells were used to target peripheral T-cell lymphomas (PTCLs) *in vitro* (141). Although it was shown that UCB-derived CD4 CAR-T cells efficiently suppressed the growth of lymphoma cells *in vitro* (with the overnight elimination rate of 38, 62 and 85% at E:T ratios of 2:1, 5:1 and 10:1 respectively) while also significantly prolonging mouse survival (>30 days vs ~20 days in controls), unfortunately, the study provided little information concerning UCB efficacy. Finally, Olbrich et al. tested the effectiveness of UCB CAR-T cells against human cytomegalovirus (HCMV)-infected cells with promising results showing high on-target effect (~30- 40% of dead target cells) and cytotoxicity *in vitro* (142). In the *in vivo* model, one week after administration, response to CAR-T cell therapy was observed in five out of eight mice, defined by significant reduction of the bioluminescent signal in relation to untreated controls. More importantly, none of all the treated mice showed adverse clinical symptoms such as loss of body weight, observable change in behavior, eczema or GvHD.

In summary, all the above-mentioned studies provided evidence of the anti-tumor efficacy of allogeneic CAR-T cells generated from UCB. However, although the feasibility of the manufacturing process as well as the anti-tumor cytotoxicity of CAR-T cells has been proved, few efficacy comparison between PB and UCB cells has been made. **Table 2** summarizes the clinical trials results, presenting information on CAR constructs as well as manufacturing details.

**Table 2:** Summary of preclinical studies investigating UCB-derived CAR T cells.

Study	Disease model	Type of study	CAR construct	Cytokines used in manufacturing process	Reference
Serrano et al., 2006	B-cell lymphoma	*In vitro* *In vivo*	CD19scFv -CD3ζ	IL-2	(130)
Huang et al., 2008	B-cell leukemia, B-cell lymphoma	*In vitro* *In vivo*	CD19scFv-4-lBB-CD3ζ	IL-2, IL-7	(134)
Micklethwaite et al., 2010	B-cell ALL	*In vitro*	CD19scFv -CD28-CD3ζ	IL-7, IL-12, IL-15	(135)
Tammana et al., 2010	B-cell leukemia, B-cell lymphoma	*In vitro* *In vivo*	CD19scFv -CD28-CD3ζ; CD19scFv-4-lBB-CD3ζ; CD19scFv-CD28-4-lBB-CD3ζ	IL-2, IL-7	(132)
Pegram et al., 2015	B-cell ALL	*In vitro* *In vivo*	CD19scFv -CD28-CD3ζ; CD19scFv -CD28-CD3ζ/IL-12; CD19scFv-4-lBB- CD28-CD3ζ; CD19scFv-4-lBB- CD28-CD3ζ/IL-12	IL-2, IL-7, IL-12, IL-15	(138)
Ma et al., 2016	AML	*In vitro*	PR1/HLA-A2scFv -CD28-CD3ζ (h8F4-CAR)	IL-2	(140)
Pinz et al., 2016	PTCL	*In vitro*	CD4scFv-CD28-4-lBB-CD3ζ	IL-2	(141)
Olbrich et al., 2020	HCMV-infected cells	*In vitro* *In vivo*	gBscFv -CD28-CD3ζ; gBscFv-4-lBB-CD3ζ;	IL-7, IL-15	(142)
Caël et al., 2022	BPDCN	*In vitro* *In vivo*	CD123scFv-CD28-4-lBB-CD3ζ	IL-7, IL-15	(126)
Liu et al., 2022	B-cell ALL	*In vitro* *In vivo*	CD19scFv -CD28-CD3ζ-T2	IL-2	(136)

ALL, acute lymphoblastic leukemia; AML, acute myeloblastic leukemia; BPDCN, blastic plasmacytoid dendritic cell neoplasm; CD, cluster of differentiation; gB, glycoprotein B; HCMV, human cytomegalovirus; IL, interleukin; PTCL, peripheral T-cell lymphoma; scFv, single-chain variable fragment; T2, Toll/interleukin-1 receptor domain of TLR2.

### 6.4 Clinical reports of UCB applications in CAR-based therapies

Following the limited but encouraging preclinical studies, there is a clear demand for additional advancements in CAR-T cell therapies utilizing UCB. We have searched the ClinicalTrials.gov database and found several registered clinical trials evaluating UCB CAR T cells (January 2025). A group from Henan Cancer Hospital is investigating the safety and efficacy of UCB CAR-T cells redirected against relapsed/refractory B-cell leukemia or lymphoma (clinical trials.gov; ID NCT03881774) (143). This phase 1 study involves patients with disease relapse following autologous CAR-T therapy and those unsuitable for autologous therapy due to the low quality of lymphocytes. Although the estimated completion date was January 2022, no results have been published yet. The group from the University College of London aims to investigate the manufacturability of allogeneic cord-blood derived T cells in a laboratory setting, evaluate their safety and efficacy of treatment in individuals with high-risk, relapsed/refractory B cell malignancies (clinical trials.gov; ID NCT05391490). Several studies are also evaluating the safety and efficacy of CB-derived CAR-NK therapies for treating various malignancies (clinical trials.gov; NCT05922930, NCT06066424, NCT05008536, NCT05703854, NCT05020015, NCT06358430, NCT05092451, and NCT05110742). For instance, the Xinqiao Hospital of Chongqing group is examining UCB-derived CAR-engineered NK cells in the treatment of patients with relapsed and refractory MM. The CAR incorporated in the NK cells facilitates the identification and elimination of MM cells by specifically targeting BCMA, a protein present on the surface of malignant plasma cells (clinicaltrials.gov; ID NCT05008536). Following the promising results of the phase 1 trial, Takeda Pharmaceuticals is currently conducting a phase 2 study of the safety and efficacy of UCB-derived NK CAR-T cells in adult patients with relapsed or refractory B-cell Non-Hodgkin Lymphoma (clinical trials.gov; ID NCT05020015). With several clinical trials ongoing, only a limited number of studies have demonstrated the safety and efficacy of CAR-NK therapy. Qian et al. evaluated the therapeutic potential and safety profile of CB-derived CAR-NK cells targeting CD19 in patients with relapsed or refractory B-cell cell lymphoma. The results demonstrated that CAR-NK cell therapy was well-tolerated, with no major adverse events such as CRS, neurotoxicity, or GvHD in none of the 9 treated patients. Moreover, the median progression-free survival was 9 months, and the complete responses were achieved in 55% of the cases, with a 58.33% overall response rate at the end of the study (clinicaltrials.gov; ID NCT05472558). Second study conducted by MD Anderson Cancer Centre has also been completed (clinical trials.gov; ID NCT03056339). The investigators enrolled 11 patients with CD-19-positive malignancies and treated them with next-generation anti-CD19 CAR NK cells engineered to express IL-15 and inducible caspase 9 safety switch (85). Of the 11 treated patients, eight (73%) had a response; of these patients, seven (four with lymphoma and three with CLL) had a complete remission, and one had remission of the Richter’s transformation component but had persistent CLL. Seven out of 11 patients achieved complete response, whereas none of the patients developed adverse events such as CRS or neurotoxicity despite HLA-mismatch (4/6 match in 9 patients). The study proved that UCB could be used as the allogeneic source for cellular therapies without requiring full HLA matching. Following the success of the Phase 1/2 results, the study progressed to an expansion phase (n = 26) and included 37 heavily pretreated patients with relapsed or refractory B cell malignancies demonstrating an ORR of 48.6% at both day 30 and day 100, with 1-year overall survival at 68% and progression-free survival at 32%. Rapid responses were observed at all dose levels: 100% of patients with low-grade NHL, 67% of patients with CLL without transformation and 41% of patients with diffuse large B cell lymphoma (DLBCL) achieved an OR. Most responses were complete responses (CRs), with 1-year cumulative CR rates of 83%, 50% and 29% for patients with NHL, CLL and DLBCL, respectively. Notably, CAR19/IL-15 cord blood units (CBU)-NK cells exhibited a comparable efficacy profile to autologous CAR19 T cells, while their safety profile was superior, showing no significant toxicities such as neurotoxicity, or GvHD and only one developed CRS (grade I). Although CAR-NK cells cannot be directly compared with CAR-T cells as NK cells possess distinguished immunogenic properties that decrease GvHD rates, CAR-NK cells present a promising, safe, and effective therapeutic option for patients with challenging B-cell malignancies (144, 145).

## 7 Cord blood bank as a source for CAR-T cell therapy

Stored in cord blood banks, UCB has established its role as an alternative source of hematopoietic stem cells for alloHSCT (146). Meanwhile, there is an increasing interest in using cord blood cells for new clinical and research applications. UCB has been regarded as an allogeneic and off-the-shelf source of NK cells as it contains relatively young and naïve NK cells, which may have greater potency and versatility in attacking target cells compared to those from adult donors (147) UCB is a rich source of progenitors and stem cells like mesenchymal stem cells (MSC), therefore UCB-derived MSCs have gained much interest for the use of potential therapeutic reasons (103, 148). Recently, it has been also proposed that a cord blood bank may function as a source for allogeneic CAR-T cell therapies (7, 126, 149).

The manufacturing of UCB CAR-T cells, as well as PB-derived CAR-T cells, consists of several key steps including T cell isolation, activation, gene modification and *ex vivo* CAR-T cell expansion (150). However, UCB-derived CAR-T cells face unique challenges due to the limited volume of UCB collections and their high nucleated red cell and mononuclear cell content which may complicate T cell isolation and processing (151). Additionally, because UCB is restricted by the small volume and low number of hematopoietic cells, *ex vivo* expansion is often required before adoptive cellular transfer (131). One remarkable advantage of UCB-derived CAR-T cells is their off-the-shelf availability, as UCB units are typically cryopreserved in UCB bank, easily accessible when needed. In contrast, autologous PB- derived T cells require patient-specific leukapheresis, leading to prolonged manufacturing and waiting time (110). What is more, allogeneic PB-derived CAR-T cells require additional genetic modifications such as elimination of the TCR receptor in order to prevent GvHD or immune rejection, a step that is not necessary in case of autologous CAR-T cells (152).

The quality of the starting material is crucial for successful CAR-T cell manufacturing, as demonstrated in autologous CAR-T therapies where baseline T cell characteristics, such as polyfunctionality, increased stemness, and reduced exhaustion, significantly impact clinical outcomes (153, 154). Similarly, for allogeneic CAR-NK cell production, donor-specific predictors of response and criteria for donor selection are vital. A study by Rezvani et al. investigated the safety and efficacy of CB-derived CAR19/IL-15 NK cells in a first-in-human phase 1/2 trial (155). The study reported day 30 overall response (OR) as the primary endpoint, with secondary objectives including day 100 response, progression-free survival, OS, and CAR19/IL-15 NK cell persistence. Among various UCB characteristics, multivariate analyses identified two key predictors of 1-year progression-free survival: a collection-to-cryopreservation time of ≤24 hours and a nucleated red blood cell (NRBC) content of ≤8 × 10^7^ cells per CBU.

Cord blood banks offer three potential approaches for CAR-T cell manufacturing. Firstly, UCB could be used to generate genome-edited next-generation CAR-T cells, as gene editing in UCB-derived CAR-T cells offers significant advantages. Due to the naïve phenotype, UCB-derived T cells are highly sensitive to gene edits that enhance persistence and proliferation, such as upregulation of memory markers like CCR7 (156). Additionally, gene editing may improve their antitumor function through increasing of cytokine production (e.g., IL-7, IL-15) and elimination of exhaustion markers like PD-1, therefore prolonging their activity (157–159).

However, due to the smaller number of CD3-positive cells compared to PB and the elimination of alloreactivity-inducing molecules in genome editing, UCB seems to be a second choice rather than the first option. The second strategy is to use UCB-banked cells as an autologous source for individuals who had their cells banked and need higher quality T cells for conventional CAR-T cell therapy. However, in this case, although T cells in the autologous cord blood unit could be expanded, overcoming technical limitations, the likelihood of an individual banking their cells (less than a few percent of the population), makes this proposal unlikely to materialize. Finally, another approach exploits the fact that in the setting of HSCT, UCB transplant requires a lower donor-recipient HLA match due to the naivety of T cells (160). In this situation, HLA-matching requires 4/6 allele complementarity (with at least one match at HLA-A, -B, and DRB1) (160). The fact that UCB units stored in the banks are HLA-typed supports the idea of generating “off-the-shelf” HLA-matched products (146). Accordingly, designing allogeneic CAR-T cells emerges as a possible application of this strategy. A population-wide bank of allogeneic CAR-T cells could be established provided that UCB-derived CAR-T cells demonstrated an acceptable safety profile in a clinical trial. Combining conventional CAR constructs with large-scale inventory would reduce costs significantly and enable product availability at request. However, such a proposal implies the calculation of a sustainable bank size. As UCB units are HLA-typed, we can estimate the required size of UCB CAR-T bank that would cover the proper population fraction at the 4/6 or higher HLA-match level. According to the analysis of the UK cord bank performed by Querol et al., a bank size of 50,000 units provides at least one donor for as much as 98% of patients (4/6 HLA match) (161). Nevertheless, decreasing the bank size has little impact on the probability of finding a 4/6 HLA-matched donor, with 10,000 unit banks still providing more than a 90% probability of donor finding (161). In the Finnish population, a bank size as small as approximately 200 units is associated with a 90% probability of finding a suitable 4/6 donor, whereas 1700 units are enough to provide 80% coverage in a 5/6 HLA setting (162). In the Korean population, a UCB donor pool required for a 95% probability of a 4/6 HLA match is estimated to be 2150 (163). For the 5/6 HLA match, the number is approximately 51,000 (163). It seems reasonable that the higher the homogeneity of the population, the lower the required size of the allogeneic CAR products. However, HLA allele and haplotype distribution within cord blood banks also play an important role, as uniformly distributed cord blood bank provides appropriate coverage while retaining a relatively small bank size (162).

Apart from biological arguments, more logistic and technical factors should be taken into account when considering cord blood banks for CAR-T production. The acquisition, processing, and depository of UCB are costly, thus relatively small percentage of the population decided on CB banking thus far, which may result in low utilization of UCB units from UCB banks (164). Additionally, the effect of long-term cryopreservation on UCB cell functionality remains unclear. Although transplantation results appear unchanged if UCB cells were cryopreserved for up to 10 years, it is yet to unravel whether longer preservation may impact UCB cell viability (165).

Moreover, both ethnic and economic challenges must be addressed to ensure fair access to UCB as a cell bank for CAR-T therapies. One of the main issues constitute lack of ethnic diversity as some of the groups are underrepresented. For instance, study by Akyurekli et al. showed that non-Caucasian ethnicity of the cord blood donor was associated with a higher risk of failing to meet banking criteria often due to lower collected volumes and reduced cell counts. This disparity can be attributed to a variety of factors, including socioeconomic barriers and lack of awareness of donation options (166). In addition, the high costs associated with collecting, processing, and storing UCB pose economic barriers, especially for lower-income families (167). Investing in cost-effective manufacturing techniques, subsidizing public UCB banking, and promoting nonprofit partnerships can help reduce the financial burden. Ethical considerations such as informed consent, donor rights, and equitable distribution of UCB-derived therapies also require clear rules to ensure transparency and trust. Addressing these issues through education, policy reform, and financial support programs will be essential to making UCB-derived CAR-T cell therapies accessible and beneficial to all patients, regardless of ethnic or economic background.

## 8 Discussion

In recent years, the field of CAR-T cell therapies has attracted the attention of researchers worldwide. It is now well-known that conventional autologous regimens have several disadvantages attributed to high costs, long time of the manufacture leading to the extended length of the “vein-to-vein” time, and unavailability for patients with low-quality T cells. Thus, numerous studies have investigated the possibilities of harnessing donor-derived CAR-T cells to address these hurdles. The allogeneic CAR-T cells gained much interest and are perceived as a promising solution to the shortcomings of the current therapies. Still, the vast majority of studies utilize the concept of genome editing that provides potent CAR-T products deprived of TCR or MHC molecules.

UCB provides multiple advantages over PB allogeneic or autologous sources for CAR-T cells. The use of UCB as a source of CAR-T cells offers accessibility as they could be transduced with conventional CAR constructs and be available at request from the CAR-T bank. The immunology of UCB T cells favors them as they are mostly composed of TSCM and TCM and therefore retained less differentiated phenotype than PB CAR-T cells (126). Additionally, UCB CAR-T cells express exhaustion markers like PD1, LAG3, or TIM3 on significantly lower levels compared to allogeneic PB-derived CAR, which translates into the capability of longer persistence *in vivo* and decreased potential risk of GVHD (125). UCB-derived CAR-T cells could be transferred both autologously to reduce post-transplant recurrence and in an allogeneic setting with fewer HLA restrictions that enable more accessible donor-recipient matching. Finally, UCB-derived CAR-T cells provide the cost-effective and sustainable strategy for utilizing cord blood banks. On the other hand, UCB poses challenges related to T cell numbers, maturity, and antigen recognition.

Yet, as the field of CAR-T cell therapy continues to advance, ongoing research is aimed at addressing these challenges and optimizing the use of UCB as a source for CAR-T cells that will overcome the challenges associated with conventional autologous or allogeneic PB-derived therapies. Although current knowledge regarding the safety and efficacy of UCB CAR-T cells is limited to preclinical and few early clinical studies, reports from the ongoing research are optimistic, and further advancements are highly awaited.
